# Housing and Support for People with Disability: Perspectives of Motor Accident, Disability and Injury Insurers across Australia and New Zealand

**DOI:** 10.3390/ijerph19159681

**Published:** 2022-08-05

**Authors:** Libby Callaway, Kate Tregloan, Natasha Layton

**Affiliations:** 1 Occupational Therapy Department, Faculty of Medicine, Nursing and Health Sciences, Monash University, Level 3, Building G, Peninsula Campus 47-49 Moorooduc Hwy, Frankston, VIC 3199, Australia; 2 Rehabilitation, Ageing and Independent Living (RAIL) Research Centre, Faculty of Medicine, Nursing and Health Sciences, Monash University, Level 3, Building G, Peninsula Campus 47-49 Moorooduc Hwy, Frankston, VIC 3199, Australia; 3 Faculty of Architecture, Building and Planning, The University of Melbourne, Parkville, VIC 3010, Australia

**Keywords:** housing, disability, injury insurance, qualitative focus group, stakeholders

## Abstract

Housing is a critical enabler of a dignified life, civic participation and the achievement of human rights. Providing appropriate housing for people who experience neurotrauma as a result of road or workplace injury, with both the assistive technology and human support required, continues however to be a policy and practice challenge. Australian and New Zealand motor accident, disability and injury insurers have high and enduring liability in this area, and their under-researched perspectives are needed to strengthen the evidence base for policy and practice development. This qualitative study utilised focus group methodology with representatives from government injury and disability insurers across Australia and New Zealand (n = 8). The study aimed to identify (a) issues and trends; (b) factors for decision making; and (c) service impacts relating to housing and support for people with disability and high daily support needs. Thematic analysis generated results across four key areas: influences on the decision to fund housing and/or support; identifying ‘good’ housing solutions; evaluating cost–benefit of housing and support investments; and developing future investment in housing and support. Findings such as those regarding decision-making, and investment, attest to the value of capturing the perspectives of this key group of stakeholders to assist to envision better housing and support for people with disability.

## 1. Introduction

A significant proportion of people who sustain traumatic injuries through road traffic or workplace accidents acquire both permanent and profound disability, often in the form of neurotrauma such as acquired brain injury (ABI) or spinal cord injury (SCI) [1]. ABI and SCI lead to significant functional impairment, along with high and enduring support needs and costs, often over a lifetime [2,3,4]. There is evidence however that with targeted skill development, accessible home and community design, effective human and technology supports, and opportunities to participate in community and build social capital, people can make independence or participation gains and housing transitions over many years [5,6,7,8].

Access to stable, supported living environments is a foundation of increased social and economic participation following neurotrauma, and is also recognised as a key determinant of health more broadly [9]. Achieving appropriate housing is challenging however due to both the breadth and complexity of health and disability-related support needs, as well as prevailing market forces (e.g., demand increasing both the housing rental and purchase market pricing; rising inflation in Australia and New Zealand, causing interest rate hikes). The provision of quality housing and coordinated supports is a policy challenge that has come into strong focus in both Australia and New Zealand. Restricted housing options mean younger people with disability continue to enter institutional settings, including residential aged care, due to limited other options [10,11].

### 1.1. Housing Rights and Disability

Australia and New Zealand became signatories to the UN Convention on the Rights of Persons with Disabilities (UNCRPD) [12] in 2007 and 2008, respectively. Article 19 of the UNCRPD recognises the right of all persons with disabilities to live in the community with choices equal to others. This includes rights to choose their place of residence and where and with whom they live; to have access to a range of in-home, residential and other community support services to support living and inclusion in the community and prevent isolation or segregation; and to have access to community services and facilities on an equal basis to persons without disabilities, and that are responsive to their needs.

However, enacting these principles within policy is complex [13]. The provision of support for people with neurotrauma differs widely, with access to injury or disability compensation and geographic location more likely to impact provision than the person’s needs and aspirations [14]. For people who do not receive compensation for the injury sustained, there have historically been few options for coordinated housing and support responses. These options are typically limited to: remaining living in the family home with informal supports into adulthood; moving to shared supported accommodation or other congregate living with people with disability; or entering a restricted range of institutional settings, including living in residential aged care as a younger person [5,15].

### 1.2. Disability and Injury Compensation and Housing Procurement

If a person’s disability or injury meets legislated criteria relating to cause and/or level of impairment sustained, they may be eligible for one of many government compensation schemes. New Zealand has a single no-fault injury insurance scheme in the longstanding Accident Compensation Corporation (ACC) (incepted in 1974) that was created with associated personal injury or ‘accident’ law reform [16,17]. A recent review of housing supports for Australians with an ABI or SCI in receipt of compensation for their injury identified varied offerings across 20 major insurance-based schemes, of which four were national [18]. From a state perspective, only Victoria has provided no-fault benefits to all injured road users, since 1987 [16]. New South Wales has had a no-fault care and support scheme for people with ‘catastrophic’ injury since 2006. In application, the NSW scheme has directly funded housing by owning and building a number of shared accommodation properties (using premium investment), and indirectly funded housing through securing suitable rental properties or modifying a claimant’s home. Tasmania has a system of direct ownership and management of a small number of supported accommodation properties provided on a no-fault basis to catastrophically injured road users but without any statutory entitlement. All other jurisdictions in Australia, as well as the New Zealand Scheme, have until recently operated with primarily common law systems that financially compensated injured workers or road users who could prove another person was at fault. Limited transitional housing, and some payment or contracting for home modifications, may have occurred upon acceptance of liability and as (in effect) an advance against future compensation.

In 2013, Australia launched an AUD $22B National Disability Insurance Scheme (NDIS), which achieved nation-wide implementation in 2020 and is transforming the way supports are provided to people with disability [14,19]. Specific to the Australian context, in 2016 it was also established that States and Territories were financially responsible for all people with a ‘catastrophic’ injury from a motor vehicle or workplace accident. From 2019, this extended to all catastrophic injury regardless of cause, forming the National Injury Insurance Scheme (NIIS) [20]. Existing schemes have therefore either changed or devolved responsibility in response to this policy reform.

ACC and NDIS policy and practice is still evolving to address scheme participants who have specialised accommodation needs. Primary ACC approaches in New Zealand include individualised plans and budgets for support at home. In Australia, the new NDIS and state-based NIIS approaches may include supported independent living payments within shared living arrangements or group homes, and specialist disability accommodation, which is specialised housing with coordinated support designed for people with extreme functional impairment, to be delivered by the market. The issue of supported housing is therefore a new consideration within these schemes and the NDIS is currently developing an overarching Home and Living policy, which is likely to change the landscape further [21].

### 1.3. Evidence of ‘What Works’

Motor accident and injury insurers have historically operated independently in their various jurisdictions to expend resources in effective and innovative ways to meet claimant need, funding service inputs to influence outcomes. There is however limited publication of scheme-specific outcomes data available in the public domain beyond statutory annual reporting, and scheme-specific outcomes data is unlikely to be published in the peer-reviewed evidence base. The advent of NDIS quarterly reporting, including key metrics around expenditure and supports, has provided some new data insights [4]. There are limited longitudinal studies on the utilisation of lump sum compensation, but anecdotally it is well understood that many people use a substantial proportion of compensation to purchase a house. The type and nature of such housing, its suitability for the person’s support needs, and long-term impact on outcomes are therefore not known.

In the Australian context, researchers have systematically informed the recent evidence base regarding housing models and features [22,23], design principles [24]; housing efficacy [25]; housing innovation by individual insurers [26,27]; housing markets [28] and stakeholder perspectives [29]. Recent mapping of the Australian funding landscape for housing for people with an acquired brain or spinal cord injury posits that publicly available information on housing and support options provided by insurers is limited, highlights ‘the need for policy makers to provide transparent information about housing entitlement’ and calls for a more unified evidence base [18].

### 1.4. Policy and Knowledge Translation

#### 1.4.1. Stakeholders and Partnerships

Relationships amongst non-government and government agencies at the service-delivery level are pivotal to enacting housing principles and policies and achievement of outcomes often relies upon ‘cooperative relations on the ground’ [30]. Joined up thinking—that is, collaborative and multilinear cross sector efforts to meet shared goals—is essential to deliver a policy agenda in a complex arena such as housing [31]. Stakeholders include individuals, groups and organisations that exist within the policy environment but are external to government, and who ‘need to be engaged for horizontal working to be effective’ [32]. Such horizontal partnerships among health system policy-makers, researchers and civil society have effectively brought about policy transformation [33]. Yet, mechanisms to connect stakeholders in the disability insurance and housing sectors appear ad hoc at best.

#### 1.4.2. Evidence-Informed Policy

Multiple factors drive and influence policy formation, and these factors include the availability of policy-relevant evidence [34,35,36]. Policy-relevant evidence is not limited to high quality scientific studies. Rather, such evidence also includes disability or insurance scheme business intelligence, and clinical practice knowledge used to answer the pragmatic and systemic concerns of policymakers [37]. The best available evidence includes relevant policies and stakeholder opinion needed by policymakers to translate efficacy research (‘can it work?’) into effectiveness research (‘does it work?’) [37].

Codified knowledge and tacit knowledge are two specific forms of evidence frequently missing from the evidence base [38]. Codified knowledge may be collected from clinical reviews, policy documents, or statistics; and tacit knowledge may be collected from face-to-face meetings or focus groups, e-mail or telephone [38]. This knowledge is often held by non-academic stakeholders, is largely unpublished and unexamined, and can be obtained by capturing the perspectives of stakeholders [39].

This research, therefore, had the overarching aim to gather perspectives from representatives of state government insurers across Australia and the Accident Compensation Commission in New Zealand in relation to housing and support policy, procurement, partnerships, and outcomes for people with disability. Three key research questions were posed: (1) what are issues and trends in housing and support policy, procurement and partnerships for Australian and New Zealand disability and injury insurers? (2) what factors inform Scheme decision making? and (3) what are the service impacts and outcomes considered relating to housing and support for people with disability and high daily support needs?

## 2. Materials and Methods

A qualitative research design was used with data collected using focus group methodology [29].

### 2.1. Inclusion Criteria

Inclusion criteria required that each participant (1) was an employee of a state-based injury insurance body in Australia or the major injury insurer in New Zealand within a senior management role with responsibility for housing policy; and (2) had nominated employment responsibility for policy, funding and/or practice relating to housing, technology and support design for people with disability in receipt of state-based injury insurance or accident compensation funded supports. Representatives meeting the study inclusion criteria were identified via public domain records, government reports, and existing government or other industry relationships of the first author. Some recommendations were also made by representatives approached for recruitment.

### 2.2. Recruitment Strategy

Potential representatives were approached in writing by the first author and provided with detail of the research project via the project explanatory statement, inclusion criteria and proposed roundtable focus group design. They were invited to consider attendance in person or via video/telephone link, or alternatively to nominate other eligible representative/s. Each participant was provided with the HREC-approved explanatory statement and signed consent to participate in the research.

### 2.3. Sample Size

Eight participants contributed to the focus group, including seven participants from six of the Australian state-based motor accident insurers and one representative from the New Zealand-based injury insurer. Three attended the focus group in person, and five attended via video link.

### 2.4. Data Collection

The roundtable focus group was held in February 2019 in a University meeting room in Melbourne, Victoria. In advance of the focus group, participants were asked to provide brief responses to questions about policy, housing and support environments; procurement and partnerships; and housing and support outcomes via an online survey (see Appendix A). Participant responses were critically reviewed by the first and second author with initial commonalities and areas of difference identified and refined into a final set of focus group questions (see Appendix B). These refined questions and a summary of respondent comments were presented to participants at the commencement of each section of the roundtable discussion. The two-and-a-half-hour focus group was audiotaped with participants’ consent. This time period included a brief introduction, conclusion and a short break. Equal time was allocated for discussion of each of the four refined focus group questions. The first and second author and another project team member co-facilitated the focus group, taking reflective notes throughout the session.

### 2.5. Data Analysis and Rigor

The audio-recording was transcribed verbatim following the focus group. The first author checked the transcript against the audiotape for accuracy. Respondent names were de-identified at the time of transcription, prior to the data being analysed using Clark and Braun’s six-stage approach to thematic analysis [40]. Analysis of the collected data was undertaken over a period of three weeks, using a 15-point checklist of criteria for good thematic analysis, to ensure enough time was allocated to this process [40]. Given the scope and scale of this project and targeted research questions, analysis was completed by hand rather than via computer-assisted analysis [41]. For the purpose of triangulation, the researchers also reviewed reflective notes taken during the focus group [42]. Emerging data patterns were identified based on the research aims, with an initial set of codes developed [43]. After coding the data, the first author examined patterns across the dataset, with consideration of preliminary themes that were both frequent but also meaningful in answering the research questions [44]. An initial five themes and 16 subthemes were identified from coding, and then examined further using both the coded data and whole data set.

To aid methodological rigor, peer checking of initial codes and themes was undertaken with the associate investigator (second author) who had co-facilitated the focus group [42]. Peer checking provided a useful opportunity to discuss potential discrepancies in the application of codes to themes. These were resolved via a negotiated consensus discussion [45]. Peer checking also allowed the first author to examine any potential for bias or influence on the research findings using reflexivity [46]. Through this process, one theme and two of the subthemes were merged through consensus discussion resulting in four key themes relating to the four research questions, with 14 subthemes. Member checking was then used, with a draft of the themes returned to study participants via email with a request to check for accuracy and resonance with their experiences and provide any feedback on the same [47]. Participants did not recommend any changes to the draft themes identified.

## 3. Results

**Figure 1:**
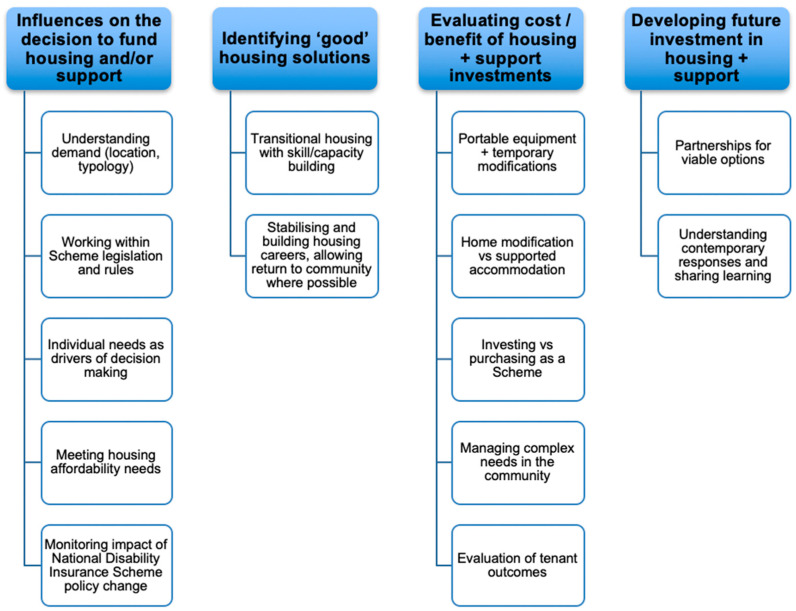
Themes identified.

Figure 1 provides an overview of the final four themes and 14 subthemes identified relating to the research questions.

Results relating to each of these themes will now be discussed.

### 3.1. Influences on the Decision to Fund Housing and/or Support

#### 3.1.1. Understanding Demand

Understanding the demand for coordinated housing and support responses in specific schemes was a key challenge, which impacted the ability of insurers to proactively plan and coordinate housing responses, and to strategize investment in existing stock, or incentivising or leading the development of new stock:

*‘We may consider building in the future, we just want to understand further about the demand.’*

Discussion covered internal data collection processes and the link to informing scheme demand and funding responses. For some insurers, location of housing for purchase had a significant impact on determining scheme investment. Several more recently established schemes discussed work underway to better understand participant profiles in order to plan housing and support funding.

#### 3.1.2. Working within Legislation and Scheme Rules

The various scheme legislative overlay and rules understandably had a significant influence on decisions regarding funding of housing and support. As noted previously, ACC approaches in New Zealand include individualised plans and budgets for support at home, but do not fund ‘housing bricks and mortar’ directly. In Australia, NDIS and state-based NIIS approaches may include legislation that accommodates funding of supported independent living payments within shared living arrangements or group homes, and the actual ‘bricks and mortar’ for specialist disability accommodation. Discussion highlighted the limitations placed on some schemes regarding their ability to purchase/supply ‘housing’ as a result of legislation which then required them to look to the existing market for purchase:

*‘We’re looking at a number of non-capital options, because of our legislation…’*

#### 3.1.3. Individual Scheme Claimant Needs as Drivers of Decision Making

Beyond the jurisdictional legislation and rules, and the impact of these on decision making, participants identified four key factors driving funding decisions: the choices expressed by scheme participants and their families; the ability to use transitional options as interim measures whilst a long-term solution was being developed; ongoing requirements of people with high daily support needs; and the capacity to return to areas where family and friendship networks existed.

Important drivers of housing and support planning were likely outcomes for scheme claimants, as well as the individual residential choices these claimants made. Study participants identified that funding decisions were linked to the support needs and goals of an individual. Providing housing and support is complex and requires coordination to be both effective, and efficient. The ability to use transitional options, that is, temporary living options as interim measures whilst a long-term solution was being developed was also seen as key.

Capacity to return people with complex needs to locations where they have family and friendship networks, share paid supports to build efficiencies, and provide accessible home environments for people with very high built design access needs were identified as drivers of funding decisions by the group:

*‘Being able to identify all of those areas that are specific for each individual person. Obviously, we’re guided by legislation, we’re guided by the schemes and what that allows us to do but looking at how we evaluate the importance and the outcomes for that I think is actually more individual specific.’*

#### 3.1.4. Meeting Housing Affordability Needs

Due to the lack of affordable housing in their jurisdiction, a number of participants acknowledged that funding decisions were at times based on a primary need for affordable housing rather than solely the need for accessible housing related to the injury or disability. This provision to meet the affordable housing gap that exists was seen as a significant challenge to manage in relation to scheme viability, and posed a barrier to further accommodation transition for such individuals:

*‘We’ve had someone in there who didn’t have high needs but who just needed accommodation and so that’s how that’s worked out.’*

#### 3.1.5. Monitoring Impact of Insurance Scheme Policy Changes

Australian focus group participants specifically identified the NDIS Specialist Disability Accommodation reform as both impacting supported housing market access for, and influencing funding decisions by, motor accident insurance schemes:

*‘We are just competing in the sector against everybody else and we have noticed some challenges [with NDIS transition]. Particularly in the supported accommodation space for our participants with extremely high needs.’*

Some participants also identified a lack of clarity at this point in time regarding the interface of state based motor accident insurance schemes and the NDIS:

*‘We’re having a look at our legislation and trying to understand how we work with the NDIS when it comes to mutual jurisdiction or mutual clients and what that might mean for our legislation’*

Focus group participants were also considering the emerging Specialist Disability Accommodation market and its impact on both the access to and range of coordinated supported housing responses for motor accident insurance scheme participants. Participants discussed the need to monitor NDIS market changes closely to inform their scheme’s strategy, particularly as the Specialist Disability Accommodation policy is implemented:

*‘We’re trying to look at what is supported accommodation and what isn’t and we’re finding it’s a very permeable space at the moment because of the new models that are coming through with the NDIS. That’s part of our housing strategy.’*

### 3.2. Identifying ‘Good’ Housing Solutions

#### Transitional Housing with Skill/Capacity Building

Transitional housing refers to tenancies of short to medium term, in settings within which supports can be adjusted. Transitional housing may be a stop gap while permanent housing is found, and may also have a therapeutic purpose in building an individual’s capacity to live as independently as possible. Participants reported that the identification of ‘good’ housing solutions for individual Scheme claimants often began with a need for a transitional option from the inpatient rehabilitation setting to facilitate return to living in the community:

*‘Independent living units are utilised … to maintain a level of independence or looking at the opportunities to increase the skills, potentially to move on…’*

This was particularly relevant when informal supports were either not available, or needed to be supplemented with paid disability supports. Transitional housing that offered skill or capacity building was seen as key in order to grow individual skills to build opportunities for further transition to more independent living:

*Stabilising and building housing careers, allowing return to community where possible*

A housing career refers to the trajectory of homes a person may have over their life, often disrupted by catastrophic injury. ‘Good’ housing and support was seen as having the potential to stabilise housing options reducing the need for a person to move across settings, and offer certainty of tenure but choice to begin to re/build housing careers. A priority in sourcing ‘good’ housing and support was the capacity to support people to return to their pre-injury community, if feasible. However, it was also noted that—for some individuals—a move away from pre-injury supports may be indicated particularly when lifestyle or social factors posed risk or challenge for the person in that previous environment:

*‘… so they’ve got very complex needs already as a result of the accident and then you don’t have stable housing to return to. That impacts on the options that are available to people’*

### 3.3. Evaluating Cost–Benefit of Housing and Support Investments

#### 3.3.1. Portable Equipment and Temporary Modifications

The group identified that the use of portable equipment (e.g., portable entry ramps) and temporary or staged modifications (e.g., modification of the bathroom as a priority area; use of portable/movable home additions for short term access needs) was a necessary and useful approach to consider as people adjusted to the outcomes of their injury and assessed their medium to longer term housing needs:

*‘One of the successes we are having is the use of our temporary modification solutions. Some of our portable bathrooms, modular ramp combinations that enable the clients to go home directly from their inpatient rehabilitation facility.’*

#### 3.3.2. Home Modification vs. Supported Accommodation

For scheme participants who require accessible housing—and were considering options that included returning to their own or the family home—re-entry to a familiar community, availability and sustainability of informal vs. paid supports, and the relationship of these factors to the cost of required home modifications were seen as key. Although the goals of the person were seen as central, the risks to the scheme associated with major home modifications that may only be accessed for short periods of time (particularly where informal supports were already strained) were closely considered. Investment in, and longevity of, home modifications and direct supports, in contrast to the cost–benefit of coordinated housing (bricks and mortar) and human support investments, was an important and at times challenging area to explore:

*‘The tipping point is often when the property might be a shack, for example, on a- like a piece of land that doesn’t have any amenities. It’s going to cost about three times the amount of the value of the home to actually modify it.’*

#### 3.3.3. Investing vs. Purchasing as a Scheme

At a scheme level, it was identified as important to try and grow access to a mixed portfolio of both affordable and accessible/adaptable supported housing options. For some schemes (e.g., New South Wales and Victoria), this included direct investment in the development of housing and support solutions as a scheme or via a property trust, whereas for others it involved purchase of existing options from other suppliers. Most often to date, such purchase had been available through disability service providers. In contrast, insurer collaboration with the housing sector was seen by some in the group as a potential new option only just beginning to be explored (see ‘Partnership for viable options’ below).

#### 3.3.4. Managing Complex Needs in the Community

The viability of creating supported housing that offered a model of care that could manage complex needs through housing in local communities was also identified as important in cost–benefit analysis. The ability for scheme claimants to share quality supports was seen as integral to evaluation of cost–benefit of housing and support investments. For example, more flexible, on-call support rather than sustained face-to-face paid supports. Specifically, managing complex continence care routines, pressure care, respiratory management and behaviours of concern were discussed. Participants discussed the importance of being able to identify ‘at risk’ groups. Identification of these groups would enable schemes to be proactive in resourcing decisions about housing and support, as well as developing future investment.

#### 3.3.5. Evaluation of Tenant Outcomes

The group discussed the need to establish evaluation criteria against which to test and consider housing and support options delivered to scheme participants:

*‘We’ve been looking at what is the minimum criteria that we want for future housing for our participants … we have been very much looking at what’s the minimum level of options for home automation in the future, what is the proximity to public—accessible public transport options. What’s the local accessibility to other facilities such as local shops or whatever it might be?’*

Evaluation and evidence of attainment of goals set by an individual, as well as positive change in home and community participation and health and wellbeing were identified as important considerations to be monitored when considering cost–benefit. The challenges in quantitatively measuring cost–benefit and outcomes with tenants was also discussed, with some schemes using qualitative approaches to understand individual experience.

### 3.4. Developing New Housing and Support Models, or Purchasing Existing Options

#### 3.4.1. Partnership for Viable Options

Growing collaborative relationships was widely seen as essential to increase viable housing and support options that delivered quality outcomes, cost benefit and scheme viability:

*‘Looking at partnerships and collaboration opportunities, particularly—well, with the private sector but I suppose more so with social housing, community housing, not for profit, the disability sector. Having a look at what opportunities might occur there.’*

Vacancy risks came about if effective and cross-sector partnerships were not well established—particularly in the case of collaborative supported housing development. One scheme was working on establishing a new, head-leasing approach with a supported housing provider (who was a disability service provider) to access housing:

*‘… we’ve got one provider working with us to establish a—I think a five-year lease with some properties—but then the idea is that the participant themselves would establish an agreement with those housing—if they agree to that particular housing option—with the housing provider and they would pay their rent to them. That would then be deducted from our leasing arrangements, so it’s almost like a pilot test case’.*

#### 3.4.2. Understanding Contemporary Responses and Sharing Learning

The group were keen to understand coordinated housing and support responses currently being delivered to social insurance claimants in Australia and New Zealand, to share learning across schemes, and to scope new responses, as this has not to date occurred. This ability to share learnings across jurisdictions was seen to benefit the range of schemes represented. Understanding contemporary responses—in contrast to the traditionally limited suite of supports consisting of group living, returning to the family home or entering residential services such as aged care—was also seen as important. Suggestions for strategies to assist this shared learning across schemes included use of existing structures, such as the Motor Accident Insurance Scheme working groups and meetings, as well as more targeted meetings (e.g., biennial) focused only on housing and support responses:

*‘I think the benefit for everyone from just seeing what each other is doing and being able to test it further and evaluate all together.’*

## 4. Discussion

This research is the first to investigate Australian and New Zealand motor accident, disability and injury insurer perspectives on housing and support policy, procurement, partnerships, and service impacts and outcomes for people with disability. Study participants identified challenges including current housing market accessibility and affordability issues, detailed a range of considerations currently being explored, and outlined strategies already established to grow the range of housing and support options available to claimants within their schemes. Key among these was access to transitional housing that offered further skill and capacity building after inpatient rehabilitation [5,16]. Often people move to more supported environments that may offer varying levels of skill building [6]. The opportunity identified in the current research to grow transitional living approaches, with a focus on skill building, was coupled with the need to develop specialised community service responses to manage people with complex or specialised needs after neurotrauma. This was particularly identified in relation to management of complex health care and behavioural support needs within community settings [18].

Australian disability policy has pivoted towards individualised budgets and market-oriented solutions, enacted through the NDIS and future establishment of a NIIS [23]. This aligns with New Zealand’s approach via the ACC. Roundtable findings demonstrated that the disability and housing policy reforms, such as the introduction of the NDIS, has had consequences for motor accident insurer activities, access to supported housing options, and considerations for future housing strategy. These findings resonate with recent literature reviewing the housing funding landscape for people with neurotrauma, which identifies a range of jurisdictional and program issues requiring policy action to resolve [18]. Close and sustained attention to impacts of policy shifts and research evidence on housing strategy for both insurers and their claimants was seen as imperative to inform further work by insurers.

It is evident from the findings that progressive work by national disability insurance and state-based motor accident and workplace injury insurers is opening new approaches for person-centred planning of both housing and support [48,49,50]. This work is however programmatic in nature, occurring within scheme silos, with varied evaluation approaches taken, and being largely unpublished. Researchers have identified the need for clearer systems [51] and call for ‘a unified, evidence-based framework to guide the funding of housing and housing support services to increase the consistency of interventions and so improve outcomes’ ([18], p. 1). Findings are therefore discussed in terms of stakeholders and partnerships, and evidence to inform policy, below.

### 4.1. Stakeholders and Partnerships

Redefining existing partnerships with disability support providers and building new collaborations across both the disability and housing sectors, was seen by the group as a key opportunity but one which would require sustained focus and effort. The introduction of the national schemes (NDIS and NIIS) means that state-based bodies have more opportunity to benefit from the sharing of learnings (as they are now impacted by the same policies) despite some state-based or jurisdictional differences [31]. Ongoing pursuit of more traditional partnerships to purchase supported housing options (e.g., with disability service providers), as well as strategies to drive new collaborations (e.g., in the social and community housing sector) or new structures (such as head-leasing options for set time periods) are opportunities that exist. The Transport Accident Commission of Victoria is a more established scheme than those in other states of Australia and has invested heavily in, and demonstrated new approaches to housing and support for claimants, by developing a property trust [29]. This research highlighted that other schemes are particularly keen to understand and learn from this new approach. The willingness to collaborate within this research, and the leadership demonstrated by some schemes to evaluate and make available project learnings [30], is a positive step towards better policy converted into real life practice for people with neurotrauma.

### 4.2. Evidence-Informed Policy

Policy which is informed by evidence is best placed to deliver equitable population health gains, value for money, and accountability in decision-making [33]. Both quantitative and qualitative evaluation approaches offer valuable avenues to understand the outcomes of investment in a range of housing and support responses. Research to evaluate social return on investment and that offers guidance regarding outcome measures to inform evaluation were identified as key necessary components currently lacking.

Understanding demand (both for typology and location of housing responses) was closely linked to an interest in developing strategies that may offer proactive and timely responses to housing and support transition. This current lack of demand data and guidance on responses was seen by some participants as a limitation on the development of proactive strategies, potentially leading to vacancy risk. Understanding the characteristics of claimants in relation to their housing and support response requirements was of substantial interest, and relates to work underway within various schemes to describe characteristics and needs of subgroups of claimants [52].

Strategies to more effectively collect and examine internal scheme data were identified as necessary. This may offer evidence of cost–benefit for a range of trialed approaches, and may flag characteristics of particular claimant subgroups that could be identified early and supported to plan for housing transitions. The ability for schemes to share housing and support evaluation approaches, de-identified outcome data, and examples of innovations in development offered an opportunity that roundtable participants were keen to further explore and to continue beyond this one-off roundtable event.

### 4.3. Limitations

The study findings and discussion should be considered in light of limitations of the current research. These include the convenience-sampled representatives of the state motor accident insurance schemes and injury insurer in New Zealand, and the varying levels of management represented in the group (and associated responsibilities and experience held). Following, this research focused only on the perspectives of motor accident and injury insurer representatives in relation to housing, technology and support-design. It is anticipated that there will be varied and valuable perspectives across stakeholder groups that warrant further investigation, including perspectives of those with lived experience of disability.

## 5. Conclusions

The practice-based knowledge generated through this research suggests that the population in need of housing will be best supported if housing strategies leverage innovative housing collaborations to increase available options; deliver a range of contemporary approaches for people with varying support needs; minimise risk of housing market failure; evaluate outcomes for scheme participants, and harness best practice housing and support approaches. Practice leadership from insurers that stimulates market responses is evidently needed. This is one of the multiple factors necessary for a coordinated or ‘joined-up’ response to the housing and support needs of people living with complex conditions and the policy development that will support it.

## Data Availability

Aligned with the human research ethics committee approval for this project, the data presented in this study are not publicly available due to privacy and confidentiality of the research participants who may be identifiable from the raw data set.

## Appendix A. Online Survey

Q1. Name (optional)
Q2. My role in housing and support design, planning or delivery is as an employee of a: state based government injury or disability scheme; national disability scheme; other; please list
Q3. I have been working in this organisation for: less than 1 year; 1–5 years; More than 5 years, but less than 10 years; More than 10 years
Q4. I have been working in my current field of practice for: less than 1 year; 1–5 years; More than 5 years, but less than 10 years; More than 10 years

*Policy, housing and support environment: Please share up to 3 key observations*

Q5. Current integrated housing and support responses my organisation is involved in for people with disability include:
Q6. Current funding structures available to people with disability to secure integrated housing and support include:
Q7. Examples of strategies for shared equity or home ownership include:
Q8. The current impact of the NDIS Specialist Disability Accommodation framework/policy and market responses on housing and support options for people with disability and high daily support needs includes:

*Procurement and Partnerships: Please share up to three key observations*

Q9. Existing or emerging partnerships and procurement relationships for housing and support for people with disability include:
Q10. Strategies identified to grow strategic partnership arrangements in relation to housing and support responses include

*Housing & Support Outcomes: Please share up to three key observations*

Q11. Built design (e.g., key current issues; opportunities; examples of innovation)
Q12. Integrated technologies (e.g., key current issues; opportunities; examples of innovation)
Q13. Relationship between housing and support provision (e.g., key current issues; opportunities)
Q14. Building of social capital and community integration (e.g., key current issues; opportunities)
Q15. Some current key barriers and enablers to investing in, purchasing and/or delivering effective housing and support include:
Q16. Other important and related matters when considering housing and support strategy with people with disability and funders that should be considered include.

## Appendix B. Focus Group Questions

Question 1: What currently influences the decision to fund housing and/or support in your insurance organisation? (prompts: criteria; transition timeframes; external influences)
Question 2: How does your organisation identify ‘good’ housing solutions for participants/claimants? (prompts: housing accessibility and amenity; anticipated tenant arrangements and outcomes; housing location; support provision)
Question 3: How does your organisation evaluate cost/benefit of housing and support investments? (prompts: community integration; costs of support; skills development for independent living; wellbeing indicators)
Question 4: How might your organisation continue to develop its investment in housing and support models in the near future? (prompts: NDIS/Specialist Disability Accommodation influence; partnership/collaboration opportunities)
